# Adult Conditional Knockout of PGC-1α Leads to Loss of Dopamine Neurons

**DOI:** 10.1523/ENEURO.0183-16.2016

**Published:** 2016-09-02

**Authors:** Haisong Jiang, Sung-Ung Kang, Shuran Zhang, Senthilkumar Karuppagounder, Jinchong Xu, Yong-Kyu Lee, Bong-Gu Kang, Yunjong Lee, Jianmin Zhang, Olga Pletnikova, Juan C. Troncoso, Shelia Pirooznia, Shaida A. Andrabi, Valina L. Dawson, Ted M. Dawson

**Affiliations:** 1 Neuroregeneration and Stem Cell Programs, Institute for Cell Engineering, Johns Hopkins University School of Medicine, Baltimore, Maryland 21205; 2 Department of Neurology, Johns Hopkins University School of Medicine, Baltimore, Maryland 21205; 3 Adrienne Helis Malvin Medical Research Foundation, New Orleans, Louisiana 70130-2685; 4 Diana Helis Henry Medical Research Foundation, New Orleans, Louisiana 70130-2685; 5 Solomon H. Snyder Department of Neuroscience, Johns Hopkins University School of Medicine, Baltimore, Maryland 21205; 6 Department of Physiology, Johns Hopkins University School of Medicine, Baltimore, Maryland 21205; 7 Division of Neuropathology, Department of Pathology, Johns Hopkins University School of Medicine, Baltimore, Maryland 21205; 8 Department of Pharmacology and Molecular Sciences, Johns Hopkins University School of Medicine, Baltimore, Maryland 21205

**Keywords:** dopamine neuron, mitochondria, neurodegeneration, PGC-1α, substantia nigra

## Abstract

Parkinson’s disease (PD) is a chronic progressive neurodegenerative disorder. Recent studies have implicated a role for peroxisome proliferator-activated receptor γ coactivator protein-1α (PGC-1α) in PD and in animal or cellular models of PD. The role of PGC-1α in the function and survival of substantia nigra pars compacta (SNpc) dopamine neurons is not clear. Here we find that there are four different PGC-1α isoforms expressed in SH-SY5Y cells, and these four isoforms are expressed across subregions of mouse brain. Adult conditional PGC-1α knock-out mice show a significant loss of dopaminergic neurons that is accompanied by a reduction of dopamine in the striatum. In human PD postmortem tissue from the SNpc, there is a reduction of PGC-1α isoforms and mitochondria markers. Our findings suggest that all four isoforms of PGC-1α are required for the proper expression of mitochondrial proteins in SNpc DA neurons and that PGC-1α is essential for SNpc DA neuronal survival, possibly through the maintenance of mitochondrial function.

## Visual Abstract

**Figure:**
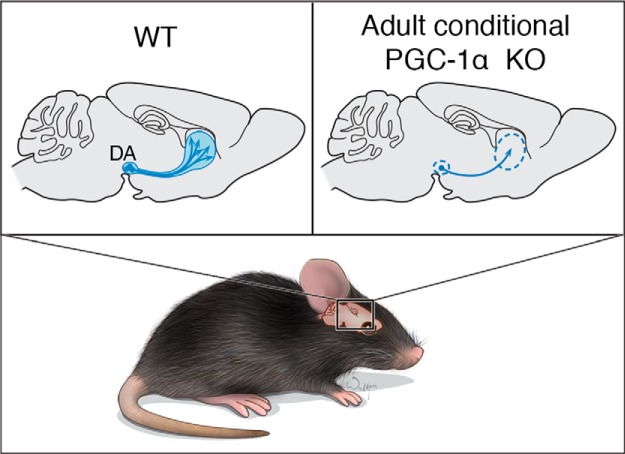

## Significance Statement

Recent studies indicate a role for peroxisome proliferator-activated receptor γ coactivator protein-1α (PGC-1α) in maintaining dopaminergic function as well as in promoting survival against toxic environments when overexpressed. It is not yet known whether PGC-1α is required for adult dopaminergic neuronal viability. To address this hypothesis, we investigated dopaminergic neuronal viability in mice following adult conditional knockout of PGC-1α and found that the loss of PGC-1α is sufficient to result in the loss of dopaminergic neurons.

## Introduction

Parkinson’s disease (PD) is the second most common chronic progressive neurodegenerative disorder. As PD progresses, patients experience motor symptoms, including tremor, slowness of movement, stiffness, loss of balance, as well as nonmotor symptoms. The changes in motor function are due to the loss of dopaminergic (DA) neurons. The factors responsible for promoting DA neuronal viability and DA neuronal death are under investigation (Lee et al., 2012; Calabresi et al., 2013). Recently, a role for peroxisome proliferator-activated receptor γ coactivator protein-1α (PGC-1α), a transcriptional coactivator, has been implicated in DA neuronal function and viability. Well studied gene targets for PGC-1α include those involved in energy metabolism, mitochondrial biogenesis, mitochondrial quality control, and mitochondrial function. Through single nucleotide polymorphism analysis, PGC-1α and brain-specific isoforms of PGC-1α have been implicated in neurodegenerative diseases, including amyotrophic lateral sclerosis, Huntington’s disease, and Parkinson’s disease (Soyal et al., 2012; Eschbach et al., 2013). In a genome-wide meta-analysis of gene expression from patients with PD and incipient Lewy body disease, genes regulated by PGC-1α are downregulated (Zheng et al., 2010), and there are PGC-1α polymorphisms that are risk factors for the development of PD (Clark et al., 2011). Additionally, in PD postmortem substantia nigra pars compacta (SNpc), there is epigenetic regulation by promoter-proximal cytosine methylation of the *PPARGC1A* gene, which encodes PGC-1α and results in the reduced expression of PGC-1α in cell models (Su et al., 2015). In murine primary cortical cultures, genetic deletion of PGC-1α leads to small increases in oligomeric α-synuclein, a key proteinaceous component of Lewy bodies, which is a hallmark feature of PD (Eschbach et al., 2015). In experimental models, PGC-1α overexpression blocks dopaminergic cell loss caused by A53T α-synuclein expression, rotenone intoxication, or parkin deficiency (Ebrahim et al., 2010). The absence of PGC-1α leads to increased susceptibility to the PD neurotoxin 1-methyl-4-phenyl-1,2,3,6-tetrahydropyridine (MPTP; St-Pierre et al., 2006). In the setting of parkin gene deletion, the elevation of PARIS, a transcriptional corepressor of PGC-1α, leads to the repression of PGC-1α. Dopaminergic loss observed due to parkin deletion and PARIS expression can be reversed by the overexpression of PGC-1α (Shin et al., 2011). Together, these studies indicate a pivotal role for of PGC-1α in dopaminergic function and a contributory role in DA neuronal viability. However, it is unknown whether PGC-1α is required for adult dopaminergic neuronal viability. To address this possibility, we investigated dopaminergic neuronal viability in mice following adult conditional knockout of PGC-1α. We report that the loss of PGC-1α results in the loss of dopaminergic neurons.

## Materials and Methods

### Animals

All experimental protocols using animals were approved by the Johns Hopkins Institutional Animal Care and Use Committee. Conditional PGC-1α knock-out mice were purchased from The Jackson Laboratory (https://www.jax.org/strain/009666).

### Immunohistochemistry

For immunohistochemistry, animals were perfused with PBS followed by 4% paraformaldehyde. Brains were postfixed with 4% paraformaldehyde and cryoprotected in 30% sucrose. Coronal sections, including striatum and ventral middle brain, were incubated with rabbit polyclonal anti-tyrosine hydroxylase (TH; catalog #NB300-109, Novus Biologicals; RRID: AB_350437). The image was visualized with 3,3'-diaminobenzidine (Sigma-Aldrich) and analyzed by Stereo Investigator software. The sections were incubated with goat polyclonal anti-green fluorescent protein (GFP) (catalog #ab6673, Abcam; RRID: AB_305643), and secondary antibodies were coagulated with Alexa fluorescent for 1 h at room temperature. Brain cell lysates were immunoblotted with mouse mono anti-PGC-1α (catalog #ST1202, Millipore; RRID: AB_2237237), rabbit polyclonal anti- succinate dehydrogenase complex, subunit A (SDHA; catalog #5839S, Cell Signaling Technology; RRID:AB_10707493), mouse mono anti-Tomm20 (translocase of outer mitochondrial membrane 20; catalog #WH0009804M1, Sigma-Aldrich; RRID:AB_1843992), monoclonal anti-β-actin-peroxidase (Sigma-Aldrich catalog #A3854; RRID:AB_262011).

### HPLC

Striatal tissue was dissected and sonicated in 0.2 ml of ice-cold 0.01 mm perchloric acid containing 0.01% EDTA and 60 nm 3,4-dihydroxybenzylamine as an internal standard. After centrifugation, 20 µl of the supernatant was injected into a column by using a Prostar-410 autosampler and isocratically eluted through a 4.6 × 150 mm C-18 reverse-phase column. The mobile phase consisted of 90 mm NaH_2_PO_4_, 1.7 mm octane sulfonic acid, 50 µm, EDTA, and 10% acetonitrile, and the flow rate was kept at 0.9 ml/min. Biogenic amines and their metabolites were detected by a dual-channel Coulchem III electrochemical detector, and the cell potential was set at E1 of −150 mV and E2 of +220 mV, and a guard cell at +350 mV. Data were collected and processed using external standards for respective amines on Clarity data acquisition software. The protein concentrations of tissue homogenates were measured using the BCA Protein Assay kit. Data were normalized to protein concentrations and expressed in nanograms per milligram protein, as previously described (Karuppagounder et al., 2016).

### Stereotaxic intranigral virus injection

For stereotaxic injection of rAAV1 overexpressing GFP or Cre-GFP, 12 6-week-old PGC-1α*^flox/flox^* mice of either sex were anesthetized with pentobarbital. An injection cannula was applied stereotaxically into the SNpc unilaterally, as previously described (Shin et al., 2011). The infusion was performed using a total of 1-2 µl of a high-titer adeno-associated virus (AAV)-GFP or AAV-Cre-GFP that was injected into each mouse. Mice were randomized for terminal analysis at 4 weeks or 6 months after injection. For Western blot analysis, SNpcs were dissected 4 weeks after viral injection, and protein samples were blotted with the primary antibodies.

### Amphetamine-induced stereotypic rotation

Four weeks after mice received the AAV1-GFP or AAV1-Cre-GFP intranigral injection into the right hemisphere, 5 mg/kg amphetamine was intraperitoneally administered to mice (Lee et al., 2013). Mice were placed into a white paper cylinder of 20 cm diameter and recorded by video for 30 min. For the last 10 min, full-body ipsilateral rotations (clockwise) were counted in 1 min intervals by a blinded viewer.

### Primer sequences for reverse transcription-PCR

The following primer sequences were used for reverse transcription (RT)-PCR: Exon B1: forward, TAC AAC TAC GGC TCC TCC TGG; Exon B2: forward, ATG GAT GAA GGG TAC TTT TGTG; Exon 3: reverse, TCA AAT GAG GGC AAT CCG TC; Exon 1: forward, TGA GTC TGT ATG GAG TGA CAT CGA GTG; mouse α4: forward, TCA CAC CAA ACC CAC AGA AA; mouse α4: reverse, CTG GAA GAT ATG GCA CAT; Human α4: forward, TCA CAC CAA ACC CAC AGA GA; Human α4: reverse, CTG GAA GAT ATG GCA CAT; Mouse Tomm20: forward, TGC TCT AAT TCC CAA GTA CTG G; Mouse Tomm20: reverse, GCA TTT GAT CCT GTA AGC TGC; Mouse SDHA: forward, ACA TTC GAC AGG GGA ATG G; Mouse SDHA: reverse, CCA AAG TAA CCT TGC CAG TC; GAPDH: forward, TGG TCT CCT CTG ACT TCA ACA GCG; GAPDH, reverse, AGG GGT CTA CAT GGC AAC TGT GAG; Mouse GAPDH: forward, CTA CAC TGA GGA CCA GGT TGT C, reverse, GTT ATT ATG GGG GTC TGG GAT GG.

### Statistical analyses

All quantitative data are expressed as the mean ± SEM. Statistical significance was determined by Student’s *t* test. Respective *p* values are indicated in the figure legends.

## Results

**Figure 1.**
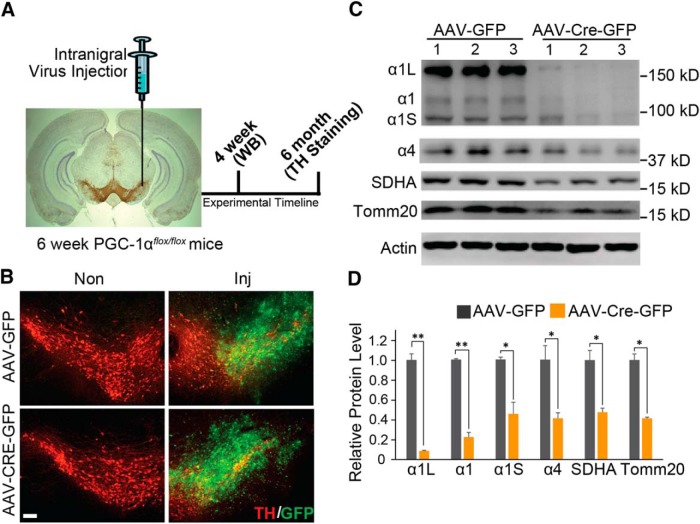
Adult conditional gene deletion of PGC-1α. ***A***, Experimental schematic of stereotaxic intranigral virus injection and experimental time line. ***B***, Representative GFP and TH-positive immunostaining of midbrain section from SNpc of PGC-1α*^flox/flox^* mice injected with AAV-GFP or AAV-Cre-GFP 6 months after the injection of virus. Scale Bar, 100 µm. ***C***, Immunoblots of PGC-1α, SDHA, Tomm20, and β-actin 4 weeks after stereotactic delivery of AAV-GFP and AAV-Cre-GFP into PGC-1α*^flox/flox^* mice; *n* = 3/group. ***D***, Quantification of ***C*** normalized to β-actin; *n* = 3/group. *p < 0.05, **p < 0.005. Inj, Injected.

Since studies suggest that there is significant compensation in the dopaminergic neuronal system when genes are deleted or overexpressed in the germline and thus during development of the dopaminergic system (Dawson et al., 2010; Shin et al., 2011), we chose to conditionally delete PGC-1α. To avoid potential developmental compensation, exons 3–5 of PGC-1α were deleted in 6-week-old PGC-1α*^flox/flox^* mice (Lucas et al., 2012) by ventral midbrain stereotaxic injection of a GFP-fused Cre recombinase AAV (AAV-Cre-GFP). Ventral midbrain injection of AAV expressing GFP (AAV-GFP) in PGC-1α*^flox/flox^* mice served as a control (Fig. 1A). Four weeks after injection, effective transduction was assessed by GFP expression, which demonstrates robust colocalization with TH-containing neurons within the substantia nigra (Fig. 1B). Transduction with AAV-CRE-GFP leads to a 78.0% loss of the PGC-1α1 isoform and a 59.1% loss of the PGC-1α4 isoform compared with the control AAV-GFP-injected mice (Fig. 1C,*D*). Immunoblot analysis also reveals the reduction of two additional isoforms of PGC-1α—a 100 kDa isoform that is reduced by 54.9% and a 160 kDa isoform that is reduced by 91.5% following AAV-CRE-GFP (Fig. 1C,*D*). The reduction in the 100 and 160 kDa bands following conditional knockout of PGC-1α suggests that the 100 and 160 kDa bands represent unrecognized isoforms of PGC-1α in the brain. Since PGC-1α contributes to the regulation of mitochondrial biogenesis (Scarpulla, 2011), mitochondrial markers for biogenesis were assessed. Accompanying the loss of the PGC-1α isoforms is a 52.5% reduction in mitochondrial protein SDHA and a 58.7% reduction in TOMM20 (Fig. 1C,*D*). Together, these data show that we achieved a conditional deletion of PGC-1α accompanied by the loss of mitochondrial markers for biogenesis.

**Figure 2.**
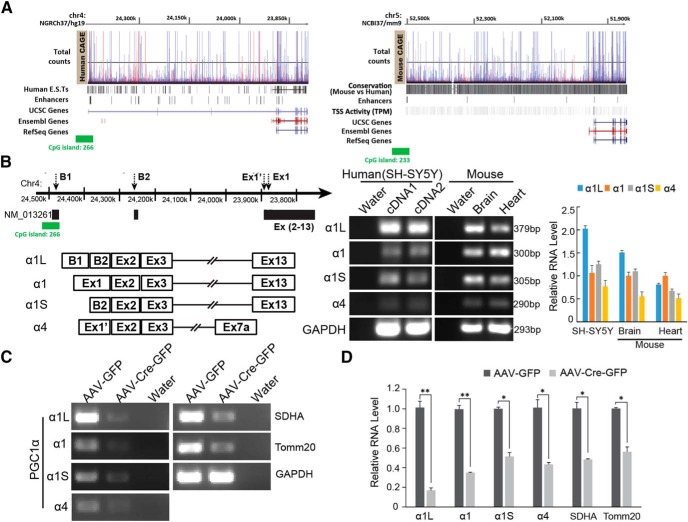
PGC-1α isoforms. ***A***, The illustration of PGC-1α new alternative exons and known exons in RefSeq/Ensembl/UCSC genome references. Human ESTs and mouse TSS with enhancer signals derived from >1000 human and mouse primary cells, cell lines, and tissues were mapped using CAGE. ***B***, Diagram and PCR products show four different PGC-1α isoforms from SH-SY5Y and mouse brain and heart. ***C***, RT-PCR for four PGC-1α isoforms, SDHA, Tomm20, and GAPDH RNA 4 weeks after stereotactic delivery of AAV-GFP or AAV-Cre-GFP into PGC-1α^flox/flox^ mice; *n* = 3/group. ***D***, Quantification of ***C*** normalized to GAPDH; *n* = 3/group. *p < 0.05, **p < 0.005.

Our observations of the reduction in the additional 100 and 160 kDa PGC-1α bands following conditional deletion suggest that there may be unrecognized genomic complexity to PGC-1α than previously thought. The genomic complexity of the protein-coding gene for PGC-1α (Human-GRCh38/hg38; chr4:23,770,000-24,475,000 and Mouse-GRCm38/mm10; chr5:51,450,000-52,120,000) was revealed by cap analysis gene expression (CAGE) dataset from the FANTOM genomics consortium (phases 1–5) through hubs on the University of California, Santa Cruz (UCSC) Genome Browser (Brett et al., 2002; Forrest et al., 2014). Expressed sequence tags (ESTs) from humans and transcription start site (TSS) activity from mice coupled with the enhancer signal indicate that PGC-1α is structurally dynamic in both humans and mice, and thus that there may be additional isoforms of PGC-1α in addition to those previously investigated (Ruas et al., 2012; Fig. 2A). To determine whether the 100 and 160 kDa bands that we observed are alternate PGC-1α isoforms, we designed PCR primers for two of these alternative isoforms that are conserved between mouse and human to determine whether the message coding for these isoforms exists in mouse brain or mouse heart or the human SH-SY5Y neuroblastoma cell line. Two of these isoforms, which we designate PGC-1α1L and PGC-1α1S corresponding to their transcript size, are detected by RT-PCR (Fig. 2B). PGC-1α1 and PGC-1α4 are also detected in both mouse brain and heart and in SH-SY5Y cells (Fig. 2B). The PGC-1α1L codes for a PGC-1α isoform with a predicted molecular weight of 160 kDa and the PGC-1α1S codes for a PGC-1α isoform with a predicted molecular weight 100 kDa are consistent with our immunoblot results. Corresponding to the reduction of PGC-1α protein in the conditional knockout of PGC-1α, we observed an 83.0% decrease of PGC-1α1L RNA, a 65.1% decrease of PGC-1α1 RNA, a decrease of 48.6% PGC-1α1S RNA, and a 56.7% decrease of PGC-1α4 RNA in the conditional knockout of PGC-1α (Fig. 2C,*D*). Accompanying the decrease of the RNA of PGC-1α isoforms is a 51.7% reduction in SDHA RNA, and a 43.7% reduction in TOMM20 RNA (Fig. 2C,*D*).

**Figure 3.**
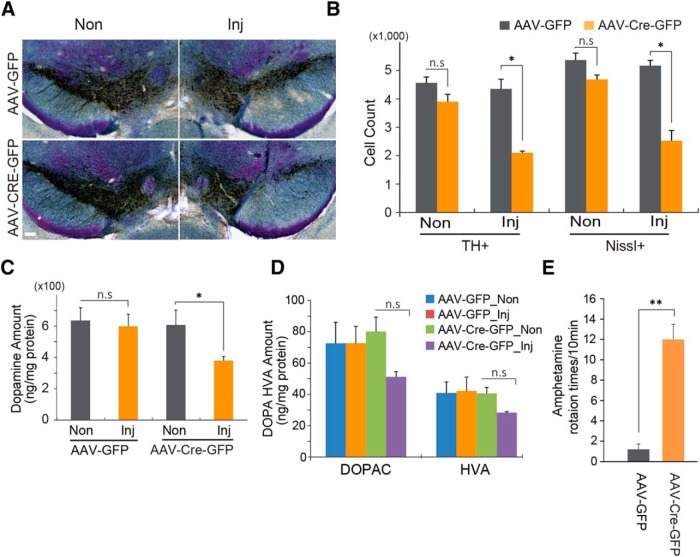
Gene deletion of PGC-1α leads to the loss of dopamine neurons in the SNpc. ***A***, Representative TH immunohistochemistry and Nissl staining of midbrain sections from SNpc of PGC-1α*^flox/flox^* mice injected with AAV-GFP or AAV-Cre-GFP 6 months after the injection of virus. Scale bar, 100 µm. ***B***, Stereological assessment of TH- and Nissl-positive neurons in the SNpc of PGC-1α*^flox/flox^* mice injected with AAV-GFP or AAV-Cre-GFP (*n* = 3/group). ***C***, HPLC assessment of the striatal content of dopamine. ***D***, HPLC assessment of the striatal content of dopamine metabolites DOPAC and HVA. ***E***, Amphetamine-induced ipsilateral rotations (*n* = 3/group). Data are expressed as the mean ± SEM. *p < 0.05, **p < 0. 005. Inj, Injected; n.s, not significant.

To determine whether PGC-1α is important in the survival of dopamine neurons, dopaminergic neuronal viability was assessed in the SNpc of adult conditional PGC-1α knock-out mice. Conditional knockout of PGC-1α leads to a 51.7% reduction in tyrosine hydroxylase-positive dopaminergic neuron and a 51.2% reduction in Nissl-stained neurons 6 months after stereotaxic injection of AAV-CRE-GFP into the ventral midbrain of PGC-1α*^flox/flox^* mice compared with the PGC-1α*^flox/flox^* mice injected with control AAV-GFP (Fig. 3A,*B*). Accompanying the loss of dopamine neurons is a 36.6% reduction in dopamine in the striatum, as determined by HPLC (Fig. 3C) and a trend toward a reduction in the dopamine metabolites 3,4-dihydroxyphenylacetic acid (DOPAC) and homovanillic acid (HVA; Fig. 3D). Since the AAV injection is unilateral, amphetamine-induced rotation was monitored to assess the functional loss of dopamine neurons. The AAV-Cre-GFP-injected mice exhibit robust ipsilateral turning, while the AAV-GFP-injected mice do not rotate (Fig. 3E).

**Figure 4.**
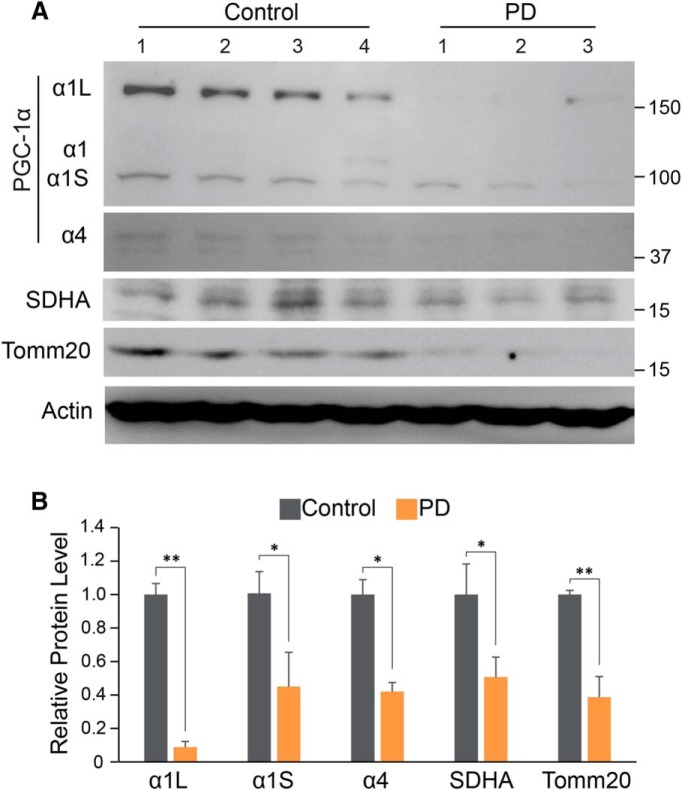
Reduction of PGC-1α isoforms, SDHA, and Tomm20 in PD. ***A***, Immunoblots of PGC-1α, SDHA, Tomm20, and β-actin in SN of PD mouse compared with an age-matched control. ***B***, Quantitation of the immunoblots in ***A*** normalized to β-actin: Control, *n* = 4; PD mouse, *n* = 3. *p < 0.05, **p < 0.005.

The levels of PGC-1α isoforms were monitored in human postmortem substantia nigra from PD and control patients (Table 1, Fig. 4). The 160 kDa PGC-1α isoform is reduced by 91.2%. The PGC-1α1 isoform was not detectable in human postmortem tissue. We cannot know whether this lack of detection is due to the specificity of the antibody or to the low abundance of this isoform coupled with the age of the tissue. The PGC-1α4 isoform is reduced by 44.7%. Accompanying these reductions in PGC-1α isoforms is a reduction in the mitochondrial proteins associated with mitochondrial biogenesis by 49.3% in SDHA and a 61.3% reduction in TOMM20 (Fig. 4A,*B*). Together, these data indicate that there is a reduction, in the human condition, in PGC-1α expression that is reflected in the loss of markers associated with mitochondrial biogenesis, suggesting that there is mitochondrial dysfunction in PD substantia nigra.

**Table 1:** Human postmortem tissue used for immunoblot analysis in Figure 4

Final diagnosis	Age (years)	Sex	Race	PMD	Tissue
Control	71	M	A	24	SN
Control	71	M	W	14	SN
Control	80	F	W	6	SN
Control	83	M	W	21	SN
PD, PD with dementia	76	M	W	29	SN
PD	72	M	W	15	SN
PD + dementia	81	F	W	13	SN

F, Female; M, male; A, African American; W, white; SN, substantia nigra; PMD, post-mortem delay.

## Discussion

The major finding of this study is the observation that adult conditional knockout of PGC-1α isoforms leads to a loss of dopamine neurons in the substantia nigra. We also identify two brain-enriched isoforms of PGC-1α that we denote PGC-1α1L (160 kDa) and PGC-1α1S (100 kDa). Importantly, these two isoforms are significantly reduced in human postmortem PD substantia nigra. Future studies will be required to understand the regulatory mechanisms of the alternative PGC-1α isoforms identified here.

A potential role for PGC-1α in PD was first suggested by the observation that germline deletion of PGC-1α increased the susceptibility of mice to the neurotoxin MPTP, which is used to selectively produce DA neurotoxicity (St-Pierre et al., 2006). However, the absence of the degeneration of dopamine neurons in the germline PGC-1α knock-out mice suggested that PGC-1α was not required for the survival of dopamine neurons. Moreover, the overexpression of PGC-1α was shown to impair dopaminergic function and increase the susceptibility of dopamine neurons to MPTP (Ciron et al., 2012; Clark et al., 2012), raising the idea that PGC-1α might be toxic to dopamine neurons. The notion that PGC-1α was not required for the survival of dopamine neurons was challenged by the discovery of parkin interacting substrate (PARIS; Shin et al., 2011). Adult conditional knockout of parkin leads to a progressive loss of dopamine neurons that is dependent upon PARIS repression of PGC-1α (Shin et al., 2011; Siddiqui et al., 2015, 2016; Stevens et al., 2015). PARIS selectively represses PGC-1α in the substantia nigra, leading to the loss of dopamine neurons due to the absence or inactivation of parkin. Consistent with the notion that PGC-1α plays a role in the loss of dopamine neurons due to parkin inactivation is the observation that the overexpression of PGC-1α prevents the loss of dopamine neurons due to the absence of parkin or PARIS overexpression. Thus, the failure to observe the loss of dopamine neurons in the germline deletion of PGC-1α is likely due to developmental compensation. Our observations of the detrimental effect of adult conditional knockout of PGC-1α in dopamine neurons supports the idea that PGC-1α is required for the survival of dopamine neurons (Mudò et al., 2012; Ciron et al., 2015).

The lack of degeneration of dopamine neurons following germline deletion of PGC-1α is likely due to compensatory mechanisms. It is well known that the rodent dopaminergic system possesses strong developmental compensatory mechanisms (Golden et al., 2013). For instance, the deletion of glial cell-derived neurotrophic factor (GDNF) during the critical period of dopamine neuronal development does not lead to the loss of dopamine neurons, whereas adult knockout of GDNF leads to a progressive loss of dopamine neurons (Granholm et al., 2011). In addition, germline deletion of parkin fails to lead to the loss of dopaminergic neurons, whereas adult conditional knockout of parkin leads to a progressive loss of dopamine neurons similar to the adult conditional knockout of PGC-1α (Shin et al., 2011). Moreover, adult deletion of the LIM homeodomain transcription factors Lmx1a and Lmx1b leads to the loss of dopamine neurons (Doucet-Beaupré et al., 2016).

In sporadic PD, genes regulated by PGC-1α are significantly reduced (Zheng et al., 2010; Shin et al., 2011). PGC-1α regulates genes involved in mitochondrial respiration and mitochondrial antioxidant defense, as well as mitochondrial biogenesis (Dabrowska et al., 2015). Here, we observed a significant reduction in the levels of mitochondrial proteins involved in biogenesis, SDHA and TOMM20, suggesting that the reduction in PGC-1α levels leads to a reduction in mitochondrial biogenesis. Similar observations have recently been reported in adult conditional parkin knock-out mice and mice overexpressing PARIS due to the reduction of PGC-1α levels (Stevens et al., 2015). Together, these studies indicate that the maintenance of PGC-1α levels in PD could offer neuroprotective therapeutic opportunities in PD (Zheng et al., 2010; Clark et al., 2011; Shin et al., 2011; Eschbach et al., 2015; Su et al., 2015).

## Synthesis

The decision was a result of the Reviewing Editor Julie Andersen and the peer reviewers coming together and discussing their recommendations until a consensus was reached. A fact-based synthesis statement explaining their decision and outlining what is needed to prepare a revision is listed below. The following reviewers agreed to reveal their identity: Jörg Schulz, Bobby Thomas

Reviewer #1:

In this study, the authors investigated the role of PGC1 α in dopaminergic neurons by inducing AAV-Cre-mediated ablation of the floxed PGC1α gene in adult mice. They demonstrate the successful deletion of four PGC1α isoforms, two of which were identified as novel. They also show that deletion of the PGC1α gene leads to the degeneration of TH+ neurons of the substantia nigra and a concomitant decrease in striatal dopamine. Amphetamine-induced rotation behavior in these mice supports the dysfunction of dopaminergic neurons. Finally, the authors find reduced expression of PGC1α isoforms in human post mortem tissue (SN).

Overall, this is an excellent study introducing a new mouse model that is appealing to both the PD field and a wider audience. Most remarkably adult-onset induction of PGC-1α induces spontaneous degeneration of dopaminergic neurons.

Minor Comments:

1. The results section is quite terse and in several instances there should be more elaborate explanations and conclusions.

2. Why were SDHA and Tomm20 picked? Please give rationale.

3. "AAV-GFP injected mice do not rotate" (Fig 3E not D)

5. The observation "the PCG1 α isoform was not detectable" is left uncommented. Is this an antibody-related problem?

6. "no significant change was observed for COXIV (data not shown)" is a puzzling final sentence in the results part: Why is it relevant to mention this result if the data is not shown?

7. The statement "two brain-enriched isoforms of PCG1α" in the discussion section is uncorroborated as the results in Fig 2B are not quantitated.

8. Legend in Fig 1B: Please show representative images of both the AAV-GFP and AAV-Cre-GFP. Also scale bar is missing.

9. Fig 1C: kD is missing

10. Fig 2C: This does not look like a real time PCR using ΔΔCt analysis but rather end-point RT-PCR. Please revise figure legend.

11. Fig 3A: Scale bar is missing

Reviewer #2:

This study demonstrates that postnatal deletion of PGC-1alpha using a AAV-mediated cre using the PGC-1alpha floxed mice impairs expression of mitochondrial proteins and results significant loss of of midbrain DA neurons. The authors also showed that four different isoforms of PGC-1alpha are expressed in the mice brain and all of them seem to be required for the survival of postnatal DA neurons. This is an excellent manuscript where the experiments are carefully done and technically sound. The methods and experimental details are well described and the results confirm several of the previously published data on PGC-1alpha in the survival of DA neurons cited by the authors.
